# Grossesse hétérotopique à la clinique Universitaire de Gynécologies et d'Obstétrique du Centre National Hospitalier et Universitaire Hubert Koutoukou Maga du Bénin: à propos d'un cas de grossesse quadruple; 3 fœtus intra utérins et 1 fœtus abdominal

**DOI:** 10.11604/pamj.2015.20.394.5419

**Published:** 2015-04-22

**Authors:** Christiane Tshabu Aguemon, Justin Denakpo, Benjamin Hounkpatin, Lehila Bagnan Tossa, Sosthène Adisso, Jeanne Sacca, José de Souza

**Affiliations:** 1 Clinique Universitaire de Gynécologie et d'obstétrique du CNHU-HKM, Bénin; 2 Hôpital Mère Enfant, Lagune, Bénin; 3 Service de Néonatologie du CNHU-HKM, Bénin; 4 Service de Cardiologie du CNHU-HKM, Bénin

**Keywords:** Grossesse hétérotopique quadruple, césarienne, insuffisance cardiaque, quadruple heterotopic pregnancy, Caesarean, heart failure

## Abstract

La grossesse quadruple hétérotopique est exceptionnelle. La revue de la littérature en parle très peu. Les auteurs rapportent 1 cas de grossesse hétérotopique quadruple développée jusqu’à 34 semaines d'aménorrhée (3 fœtus intra utérins et 1 fœtus abdominal). Les fœtus intra utérins étaient diagnostiqués à l’échographie alors que le fœtus intra abdominal était de découverte opératoire. 3 jumeaux vont décéder en post-partum immédiat et un jumeau va survivre avec un bon développement psychomoteur. Le suivi médical de la mère en post partum a vu se développer une insuffisance cardiaque globale secondairement maitrisée avec un traitement médical.

## Introduction

La grossesse hétérotopique encore appelée grossesse combinée est une grossesse extrêmement rare. Il s'agit de grossesses gémellaires bi-ovulaires dont l'une des nidations se fait dans la cavité utérine et l'autre en situation ectopique. La fréquence de cette association ne cesse de croître avec l'avènement de l'aide médicale à la procréation et des techniques d'induction à l'ovulation. Nous rapportons un cas de grossesse hétérotopique spontanée colligé à la clinique universitaire de gynécologie et d'obstétrique du CNHU-HKM de Cotonou (Bénin). Le but de ce travail était de rappeler l'existence de ce phénomène et les difficultés diagnostiques d'une telle association.

## Patient et observation

**Figure 1:**
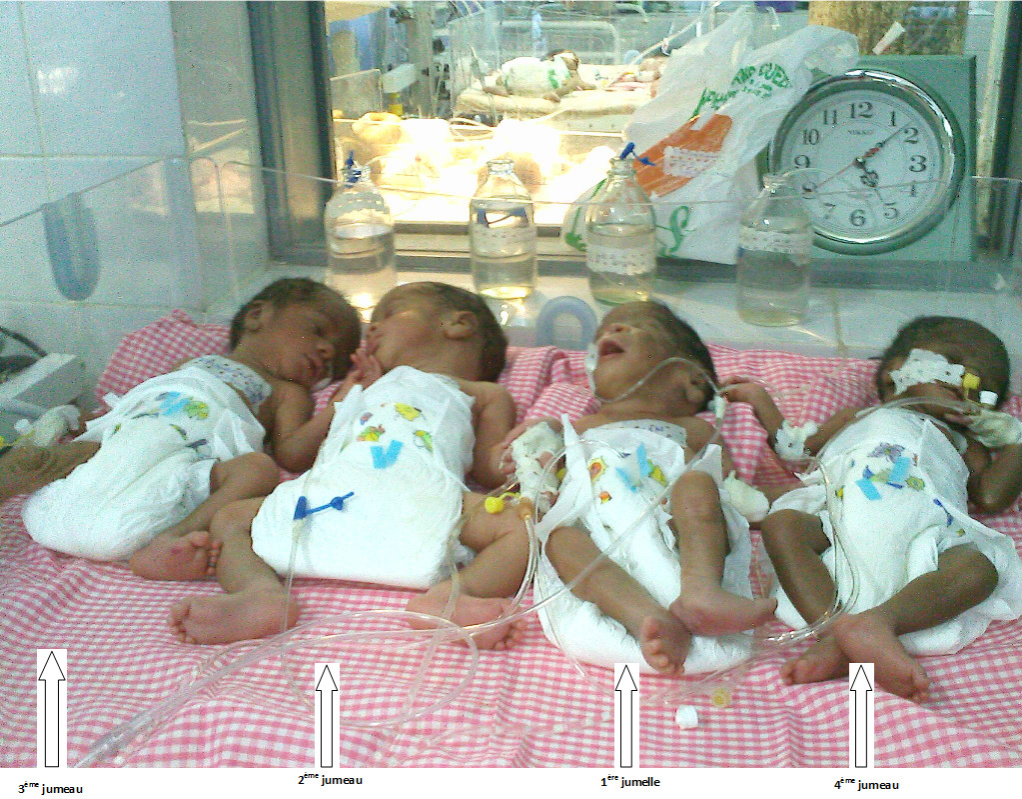
Les quatre jumeaux

**Figure 2:**
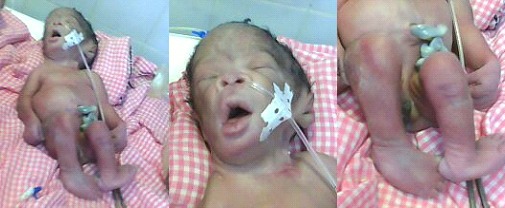
Différentes malformations externes de la jumelle

Il s'agit de madame H.E. âgée de 22 ans primigeste nullipare sans antécédent particulier ni de notion de stimulation ovarienne. Les consultations pré-natales étaient correctement suivies et tout le bilan pré-natal effectué. Lors du bilan de la confirmation de grossesse, une échographie faite à 6 semaines d'aménorrhée révélait une grossesse gémellaire bi choriale biamniotique intra-utérine. Un contrôle échographique de datation fait, avait révélé une grossesse triple évolutive intra-utérine triamniotique trichoriale au terme échographique de premier jumeau (J1) = 11semaines d'aménorrhée (SA) + 6 jours (jr), deuxième jumeau (J2) = 11SA + 1jr, troisième jumeau (J3)= 09SA avec l'ovaire droit micropolykystique. Les échographies du 2^ème^ et du 3^ème^ trimestre étaient difficiles à réaliser car les fœtus apparaissaient superposés. L’évolution de la grossesse s’était déroulée normalement jusqu’à 33 SA où la gestante avait développé un tableau de pré-éclampsie modéré qui nécessitait une hospitalisation pour un suivi pluridisciplinaire. A 34 SA, la gestante s’était plainte de douleurs sus pubiennes atroces et insomnientes, permanentes et d'intensité évolutive avec apparition brutale d'une voussure péri ombilicale. L'examen clinique suspectait une rupture utérine. Une césarienne était pratiquée en urgence avec extraction de 4 jumeaux vivants (1 fille et 3 garçons) (Figure 1). La première jumelle vivante polymalformée à développement abdominal et de découverte per opératoire pesait 1575 gr (Figure 2), les 3 jumeaux intra-utérins vivants pesaient respectivement 1980 gr, 1430gr et 1400 gr.

**Figure 3:**
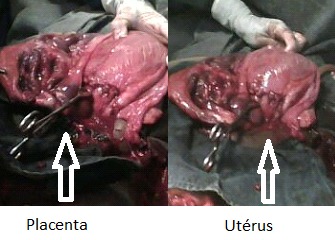
Placenta intra abdominale de la 1^ère^ jumelle

Le placenta de la grossesse abdominale était laissé en place car implanté dans le ligament large droit avec une extension sur le coecum et l'omentum (Figure 3). Une délivrance manuelle immédiate des trois placentas intra utérins était effectuée. Les quatre nouveau-nés ont été transférés dans le service de néonatologie du centre national hospitalier universitaire-HKM (CNHU-HKM) et la mère admise aux soins intensifs de la clinique universitaire de gynécologie obstétrique (CUGO) où une cure de méthotrexate (50 mg par jour pendant 3 jours) était administrée pour intensifier la lyse du placenta intra abdominal.

Sur le plan évolutif, 3 jumeaux étaient décédés successivement à 7 jours de vie (2^e^ jumeau), à 10 jours de vie (3^e^ jumeau) et à 14 jours de vie (1^ère^ jumelle) de détresse respiratoire. Le 4^e^ jumeau est vivant, aujourd'hui âgé de trois ans avec un bon développement psychomoteur.

La mère a développé une cardiopathie avec insuffisance cardiaque globale à partir du 6^e^ mois après l'accouchement et régulièrement suivie sous traitement médical anti hypertensif (Furosémide 2 comprimés par jour, Vastarel 1 comprimé par jour, Captopril 1 comprimé par jour Sintrom 1 comprimé par jour) jusqu’à ce jour. Un contrôle du placenta laissé dans la cavité abdominale au scanner, après un an, avait conclut a un nodule intra ligamentaire droit. Elle est tombée enceinte au bout de deux ans de suivi mais cela avait nécessité une interruption médicale de grossesse à 12 SA car les chiffres tensionnels étaient devenus instables et incontrôlables.

## Discussion

Le premier cas de grossesse hétérotopique a été découvert en 1761 [1] lors de l'autopsie d'une femme au troisième mois de grossesse et le second un siècle plus tard [2]. La revue de la littérature traite très peu les cas de grossesse hétérotopique évolutive au-delà de 7 SA. En cas d'association confirmée, la cœlioscopie permettait l’élimination de la grossesse ectopique laissant évoluer la grossesse intra utérine [3–6]. Dans notre cas clinique, le diagnostic de la grossesse ectopique a été fait en per opératoire; ce qui faisait penser à certaines difficultés lors des différentes échographies faites pendant la gestation. Notre difficulté diagnostique parait se reposer sur le nombre de fœtus intra utérins. Malgré tout, le suivi était bon et tous les nouveau-nés sont sortis vivants après la césarienne. Même si la première jumelle est née polymalformée, malformation probablement liée à sa position abdominale, son décès et celui des deux autres peuvent être liés à l'indigence du plateau technique et éventuellement à des malformations internes qui, malheureusement, n'ont pu être déterminées chez la jumelle, les réalités béninoises interdisant l'autopsie des jumeaux. Les deux autres jumeaux ont développé la maladie des membranes hyalines à cause de la prématurité. La mère n'a pas été épargnée puisque à l'accouchement et même au contrôle cardio-vasculaire un mois après l'accouchement, tout paraissait normal mais 6 mois plus tard elle a développé une insuffisance cardiaque globale préjudiciable pour sa procréation et qui l'oblige à un traitement continu. L'interruption thérapeutique d'une deuxième grossesse, deux ans après son 1er accouchement, était obligatoire. La prise en charge des grossesses ectopiques par l'administration du méthotrexate comme antimitotique est connue; ceci a permis la réduction du placenta laissé dans la cavité abdominale en un nodule intra ligamentaire droit.

## Conclusion

La grossesse hétérotopique menée jusqu'au troisième trimestre de la grossesse est exceptionnelle. De notre observation, il ressort que la survie materno-fœtale des femmes porteuses de grossesse à haut risque passe par l'amélioration du plateau technique et que l'examen cardiaque régulier doit faire partie intégrante lors des examens prénataux.
